# Frailty in rheumatoid arthritis and its relationship with disease activity, hospitalisation and mortality: a longitudinal analysis of the Scottish Early Rheumatoid Arthritis cohort and UK Biobank

**DOI:** 10.1136/rmdopen-2021-002111

**Published:** 2022-03-15

**Authors:** Peter Hanlon, Fraser Morton, Stefan Siebert, Bhautesh D Jani, Barbara I Nicholl, Jim Lewsey, David McAllister, Frances S Mair

**Affiliations:** 1 General Practice and Primary Care, University of Glasgow Institute of Health and Wellbeing, Glasgow, UK; 2 Institute of Infection, Immunity & Inflammation, University of Glasgow, Glasgow, UK; 3 Health Economics and Health Technology Assessment, University of Glasgow Institute of Health and Wellbeing, Glasgow, UK; 4 Public Health, University of Glasgow Institute of Health and Wellbeing, Glasgow, UK

**Keywords:** rheumatoid arthritis, epidemiology, patient reported outcome measures

## Abstract

**Objective:**

To assess the prevalence of frailty in rheumatoid arthritis (RA) and its association with baseline and longitudinal disease activity, all-cause mortality and hospitalisation.

**Participants:**

People with RA identified from the Scottish Early Rheumatoid Arthritis (SERA) inception cohort (newly diagnosed, mean age 58.2 years) and UK Biobank (established disease identified using diagnostic codes, mean age 59 years). Frailty was quantified using the frailty index (both datasets) and frailty phenotype (UK Biobank only). Disease activity was assessed using Disease Activity Score in 28 joints (DAS28) in SERA. Associations between baseline frailty and all-cause mortality and hospitalisation was estimated after adjusting for age, sex, socioeconomic status, smoking and alcohol, plus DAS28 in SERA.

**Results:**

Based on the frailty index, frailty was common in SERA (12% moderate, 0.2% severe) and UK Biobank (20% moderate, 3% severe). In UK Biobank, 23% were frail using frailty phenotype. Frailty index was associated with DAS28 in SERA, as well as age and female sex in both cohorts. In SERA, as DAS28 lessened over time with treatment, mean frailty index also decreased. The frailty index was associated with all-cause mortality (HR moderate/severe frailty vs robust 4.14 (95% CI 1.49 to 11.51) SERA, 1.68 (95% CI 1.26 to 2.13) UK Biobank) and unscheduled hospitalisation (incidence rate ratio 2.27 (95% CI 1.45 to 3.57) SERA 2.74 (95% CI 2.29 to 3.29) UK Biobank). In UK Biobank, frailty phenotype also associated with mortality and hospitalisation.

**Conclusion:**

Frailty is common in early and established RA and associated with hospitalisation and mortality. Frailty in RA is dynamic and, for some, may be ameliorated through controlling disease activity in early disease.

**Key messages**

**What is already known about this subject?**

- Frailty has been shown to be common in people with rheumatoid arthritis but its change over time and relationship with adverse clinical outcomes remain unclear.

**What does this study add?**

- Frailty in early rheumatoid arthritis is dynamic and responsive to treatment: following diagnosis and initiation of disease modifying antirheumatic drugs, the mean frailty index fell and 46% of moderate frailty individuals transitioned to a mildly frail or robust state.
- Frailty, by two contrasting measures, was associated with greater risk of all-cause mortality and hospitalisation.

**How might this impact on clinical practice or further developments?**

- Frailty may help identify people with rheumatoid arthritis at increased risk of adverse health outcomes.
- However, a label of frailty should be used with caution in people with active disease, for whom it may at least be partially reversible.
- Identification of frailty in people with rheumatoid arthritis should inform implementation of broad multidisciplinary assessment and intervention and focus on reversible factors.

## Introduction

Frailty describes a state of increased vulnerability to adverse health outcomes caused by reduced physiological reserve.^1^ Frailty is associated with age.^2^ However, it also predicts hospitalisation and death in younger people (<65 years).^1 3^ Frailty has also been found to be common in rheumatoid arthritis (RA), including in people <65 years.^4–7^ However, most studies have been small and cross sectional with only one examining associations between frailty and any clinically significant outcome such as hospitalisation.^8 9^

There are a number of different operational definitions of frailty. The most commonly implemented are the frailty index (a count of age-related health deficits)^10^ and the frailty phenotype (a specific syndrome based on a combination of low grip strength, weight loss, exhaustion, low physical activity and slow walking pace).^11^ Both measures are based on the identification of vulnerability to physiological decompensation, which distinguishes them from related concepts such as multimorbidity.^12^ Multimorbidity is associated with mortality in people with RA,^13^ however the relationship between frailty and these outcomes has not been widely explored in the context of RA.

Frailty and disease activity in RA are likely to share considerable overlap. Both the frailty phenotype^11^ and the frailty index^2 10^ share features with RA disease activity. Despite this, no study has assessed whether frailty in RA predicts clinical outcomes independently of disease activity, nor whether frailty, like disease activity, improves following treatment for RA. These questions are of clinical importance as they have implications for the optimal approach to the management of frailty in RA. Consequently, we assessed the prevalence of frailty in people with early and established RA; analysed change in frailty status in early RA in the period following diagnosis; and quantified the association between frailty and all-cause mortality and unscheduled hospitalisation.

## Methods

### Data sources

The Scottish Early Rheumatoid Arthritis (SERA) cohort is an inception cohort of people with newly diagnosed RA or undifferentiated arthritis recruited from 16 out of 17 specialist rheumatology units across Scotland.^14 15^ SERA participants in this study were recruited between March 2011 and April 2015. Participants were ineligible if they had previously received disease-modifying antirheumatic drug (DMARD) treatment for more than 4 weeks. Participants underwent a baseline assessment followed up 6-monthly follow-up visits.

UK Biobank is a population cohort study recruited between 2006 and 2010.^16^ Participants had to be registered with a general practice and live within 20 miles of one of 22 assessment centres in England, Scotland or Wales. Participants underwent a baseline assessment including a questionnaire, interview, physical measurements and biological samples.

Date of initial assessment for either dataset was taken as baseline for this analysis. SERA and UK Biobank participants consented to data linkage to national records including inpatient hospital records and mortality registers (available until April 2017 for both datasets).

### Study population: identifying RA

From the SERA dataset, we selected patients who fulfilled the 2010 American College of Rheumatology (ACR)/EULAR Classification Criteria for RA at baseline assessment.^17^

From UK Biobank, we identified participants from baseline UK Biobank assessments who had a previous diagnostic code for RA from either linked primary care records or inpatient hospital records.

### Frailty definition

#### Frailty index

In both UK Biobank and SERA, we quantified frailty using the frailty index approach, based on the cumulative deficit model of frailty developed by Rockwood and Mitnitski.^10^ A frailty index is a count of health related ‘deficits’ within an individual, calculated by summing all deficits present and dividing this by the total number of possible deficits, to give a value between 0 (no deficits) and 1 (all possible deficits). All deficits are weighted equally. Higher values indicate a greater degree of frailty.

There is a standardised method for constructing a frailty index.^18^ There is no prespecified list of deficits which must be included in the index. Rather, deficits are selected based on the variables available in a given dataset providing they meet the following criteria: (1) associated with poor health, (2) increase in prevalence with age, (3) cover a range of organ systems and (4) are neither too rare (ie, <1% prevalence) nor ubiquitous within the target population. Deficits typically include comorbidities, symptoms, functional limitations and laboratory investigations. If data for a specific deficit is missing, this deficit is excluded from the numerator and the denominator. We excluded participants with missing data for >5% of deficits.

For UK Biobank, we used the frailty index previously developed by Williams *et al*.^19^ For SERA, we constructed a frailty index based on 42 deficits (including similar comorbidities to the UK Biobank frailty index, as well as symptoms, laboratory deficits and functional measures previously used in a frailty index developed for RA clinical trials).^20^ See online supplemental appendix for full list of deficits.

10.1136/rmdopen-2021-002111.supp1 Supplementary data

The frailty index was analysed as a numerical variable. In addition, for presentation of data in tables and HRs, we categorised the frailty index into robust (0 to 0.12) and mild (>0.12 to 0.24), moderate (>0.24 to 0.36) and severe (>0.36) frailty based on the cut-points used in the electronic frailty index used in primary care within the UK.^21^

#### Frailty phenotype

For UK Biobank, we also assessed frailty using an adaptation of the frailty phenotype developed by Fried *et al*.^11^ The frailty phenotype is based on five characteristics: low hand-grip strength, self-reported exhaustion, unintentional weight loss, low physical activity and slow walking pace. People with three or more criteria are considered frail, while one or two criteria indicates ‘prefrailty’. We have previously adapted the original definitions of these criteria to UK Biobank data.^3^

SERA does not contain the necessary variables for the frailty phenotype.

### Measures

Age and sex were recorded at time of recruitment in both datasets. For UK Biobank, disease duration was estimated as the time since the first recorded diagnostic code for RA (for SERA all participants were recruited at the point of diagnosis by a rheumatologist). As time since initial diagnostic code is a proxy measure we did not attempt to differentiate early RA in UK Biobank. Socioeconomic status was based on an area-based measure (Townsend scores from linked 2001 census data in UK Biobank and Scottish Index of Multiple Deprivation in SERA).^22 23^ Both measures are based data linkage to participants’ postcodes and estimate socioeconomic status via a componsite measure of various factors (Townsend scores based on percentage unemployment, percentage car ownership, percentage home ownership and household overcrowding, Scottish Index of Multiple Deprivation based on income, employment, education, health, access to services, crime and housing).

Smoking status was categorised as current, previous or never. Alcohol intake was based on self-reported frequency of intake in UK Biobank and on self-reported weekly units in SERA.

### Outcomes

In SERA, we assessed the relationship between baseline frailty and RA disease activity, assessed using the composite Disease Activity Score in 28 joints, C reactive protein (CRP) version (DAS28) based on four factors (tender joints, swollen joints, CRP and patient global score). Physical function was assessed using the Health Assessment Questionnaire-Disability Index (HAQ-DI), and self-rated health was assessed using the visual analogue scale (0–100) from the EuroQol 5-Dimension (EQ-5D) questionnaire. DAS28, HAQ-DI and self-rated health were assessed at baseline and then at 6-monthly follow-up intervals.

In both datasets, we assessed the relationship between frailty and both all-cause mortality and all-cause unscheduled hospitalisation (defined as any admission with an ‘urgent’ or ‘emergency’ code), identified through linkage to national mortality registers and hospital records, respectively. These linked datasets record all inpatient hospital episodes and recorded deaths in either Scotland (SERA) or for the entire UK (UK Biobank). Mean follow-up was 10 years in UK Biobank and 4 years in SERA. Participants were censored at death or end of available follow-up (April 2017), whichever occurred first.

### Statistical analyses

#### Distributions of frailty

For SERA, the individual participant data are held within a secure safe-haven which only allows export of aggregate, non-disclosive data. Therefore, to allow us to describe the distribution of the frailty index, we assessed the fit of possible distributions for a frailty index (lognormal, exponential, Weibull and generalised-gamma) using the Kolmogorov-Smirnov test. The generalised-gamma distribution fitted well. These parameters were then exported from the safe-haven and used to plot the distribution of the frailty index.

For UK Biobank, we plotted the full distribution of the frailty index and described this distribution statistically.

To facilitate interpretation, we also calculated percentages of participants who were robust or had mild, moderate or severe frailty. These findings are presented as descriptive statistics only.

The frailty index distribution was summarised descriptively for each dataset separately. This is because the deficits included in each index differ, and the method used to identify RA also differed between SERA and UK Biobank.

#### Frailty and disease activity (SERA only)

For SERA, we assessed the relationship between the frailty index and activity of RA using the DAS28 score. We used generalised gamma regression to model the frailty index on age, sex and DAS28. The coefficients and variance covariance matrix from this model were then exported from the safe-haven and used to model the mean frailty index conditional on a specific age, sex and DAS28 value. We therefore modelled mean frailty for men and women index at a range of ages (30–80 years) and DAS28 values (3.2 indicating the threshold for mild disease activity, and 5.1 indicating the threshold for active disease).

#### Frailty and outcomes: serial follow-up in SERA

To assess the change in frailty index over time, we recalculated the frailty index at 6-monthly follow-up intervals. This period is concurrent with the commencement of disease-modifying treatment (reported elsewhere).^15^ We did not formally assess treatment status. As comorbidities were only assessed at baseline, we carried baseline comorbidity status forward. For all other deficits (functional measures, symptoms, and blood results) the frailty index used follow-up values. Frailty index was treated as missing where these additional values were not assessed at follow-up, in which case the previous frailty index value was carried forward. We then plotted the mean frailty index at follow-up, as well as the mean DAS28 score, mean HAQ-DI score, and mean self-rated health (using the EQ-5D visual analogue scale) at each follow-up point. Participants were excluded where data on these outcomes were missing. We assessed these outcomes over the first 2 years of follow-up.

#### Frailty and outcomes: linked healthcare data

We used negative binomial regression to model the number of urgent or emergency admissions on the frailty index (SERA and UK Biobank) and the frailty phenotype (UK Biobank only). For all-cause mortality, we used Cox proportional hazards models to model mortality on frailty index. We fit three models for each outcome. Model 1 adjusted for age, sex and socioeconomic status. Model 2 additionally adjusted for smoking status and alcohol intake. Model 3 adjusted for variables in model 2, plus DAS28 (SERA only). Incidence rate ratios (IRR) and Hazard ratios (HR), respectively, were calculated with 95% CIs. Participants with missing data for covariates were excluded from the adjusted analyses.

As a sensitivity analysis using the SERA dataset, an extended cox-PH model was used to model the effect of changing frailty index and DAS28 values on hospitalisation and mortality.

We fit models 1 and 2 using the frailty phenotype (UK Biobank only).

### Patient and public involvement

No patients were involved in this research.

## Results

In SERA, 899 participants had RA at baseline, recruited at the time of diagnosis (median symptom duration 6 months). In UK Biobank, at baseline assessment, 3605 participants had a prior diagnostic code for RA in either primary care records or inpatient hospital records. Baseline characteristics are shown in table 1.

**Table 1:** Baseline demographic characteristics stratified by frailty status

	SERA				UK biobank (RA population only)							
	Total (n=899)	Frailty index			Total (n=3605)	Frailty index*				Frailty phenotype†		
		Robust (n=303)	Mild (n=487)	Moderate/severe (n=109)		Robust (n=773)	Mild (n=2001)	Moderate (n=714)	Severe (n=109)	Robust (n=788)	Prefrail (n=1775)	Frail (n=781)
Mean age												
Years (SD)	58.3 (13.3)	56.0 (13.6)	58.5 (13.8)	63.7 (12.3)	59.5 (7.1)	59.2 (7.2)	59.5 (7.1)	59.8 (6.9)	58.2 (5.9%)	58.8 (7.1)	59.8 (7.1)	59.0 (7.0)
Sex												
Male (%)	313 (34.8)	109 (36.0)	171 (35.1)	33 (30.3)	1063 (29.5)	255 (33.2)	568 (28.4)	208 (29.1)	27 (24.8)	278 (35.3)	527 (29.7)	193 (24.7)
Female (%)	586 (65.2)	194 (64.0)	316 (64.9)	76 (69.7)	2542 (70.5)	518 (66.8)	1433 (71.6)	506 (70.9)	82 (75.2)	510 (64.7)	1248 (70.3)	588 (75.3)
SES												
Quintile 1 (deprived)	193 (21.6)	60 (19.8)	102 (21.1)	31 (28.4)	884 (24.5)	115 (15.0)	459 (23.0)	255 (35.7)	52 (47.7)	132 (16.8)	396 (22.3)	270 (34.6)
2	193 (21.6)	58 (19.1)	111 (23.0)	24(22.0)	790 (21.9)	169 (21.9)	449 (22.4)	148 (20.7)	22 (20.2)	173 (22.0)	385 (21.7)	181 (23.2)
3	174 (19.4)	62 (20.5)	90 (18.6)	22 (20.2)	698 (19.4)	161 (20.9)	391 (19.5)	129 (18.1)	15 (13.8)	167 (21.2)	336 (18.9)	142 (18.2)
4	191 (21.3)	69 (22.8)	108 (22.4)	14 (12.8)	593 (16.5)	162 (20.8)	328 (16.4)	91 (12.7)	12 (11.0)	145 (18.4)	314 (17.7)	98 (12.5)
Quintile 5 (affluent)	144 (16.1)	54 (17.8)	72 (14.9)	18 (16.5)	639 (17.7)	166 (21.4)	373 (18.6)	91 (12.7)	8 (7.3)	171 (21.7)	343 (19.3)	90 (11.5)
Missing	0	0	0	0	0	0	0	0	0	0	1	0
Smoking												
Never	323 (35.9)	115 (38.0)	167 (34.3)	41 (37.6)	1624 (45.6)	402 (52.5)	894 (45.0)	293 (41.6)	35 (32.1)	366 (46.7)	810 (45.9)	330 (42.9)
Previous	326 (36.3)	108 (35.6)	181 (37.2)	37 (33.9)	1512 (42.4)	295 (38.6)	866 (43.6)	303 (43.0)	48 (44.0)	346 (44.2)	748 (42.4)	317 (41.2)
Current	249 (27.7)	79 (26.1)	139 (28.5)	31 (28.4)	429 (12.0)	68 (8.9)	227 (11.4)	108 (15.3)	26 (23.9)	71 (9.1)	205 (11.6)	123 (16.0)
Missing	1	1	0	0	40	8	14	10	0	5	12	11
RA duration												
Median years (IQR)	–	–	–	–	6.6 (3.0–10.7)	6.2 (3.0–11.2)	7.0 (3.2–11.0)	6.2 (2.7–9.8)	6.0 (2.8–9.4)	5.9 (2.7–10.0)	6.6 (3.0–10.8)	7.0 (3.6–10.6)
Mean DAS-28												
Score (SD)	4.9 (1.3)	4.1 (1.2)	5.2 (1.1)	6.0 (1.1)	–	–	–	–	–	–	–	–
Missing	65	31	30	4								
Mean HAQ-DI												
Score (SD)	1.2 (0.8)	0.5 (0.5)	1.5 (0.6)	2.2 (0.6)								
Missing	2	2	0	0								
Mean self-rated health												
Score (SD)	55.4 (25.8)	25.9 (24.0)	50.0 (23.2)	39.2 (22.8)								
Missing	7	3	3	1								

*Eight UK Biobank participants had missing values for the frailty index and are excluded from columns stratified by frailty index.

†A total of 262 UK Biobank participants had missing data for the frailty phenotype and are excluded from columns stratified by frailty phenotype status.

DAS-28, Disease Activity Score in 28 joints; HAQ-DI, Health Assessment Questionnaire – Disability Index; RA, rheumatoid arthritis; SERA, Scottish Early Rheumatoid Arthritis; SES, socioeconomic status.

### Distributions of frailty

**Figure 1:**
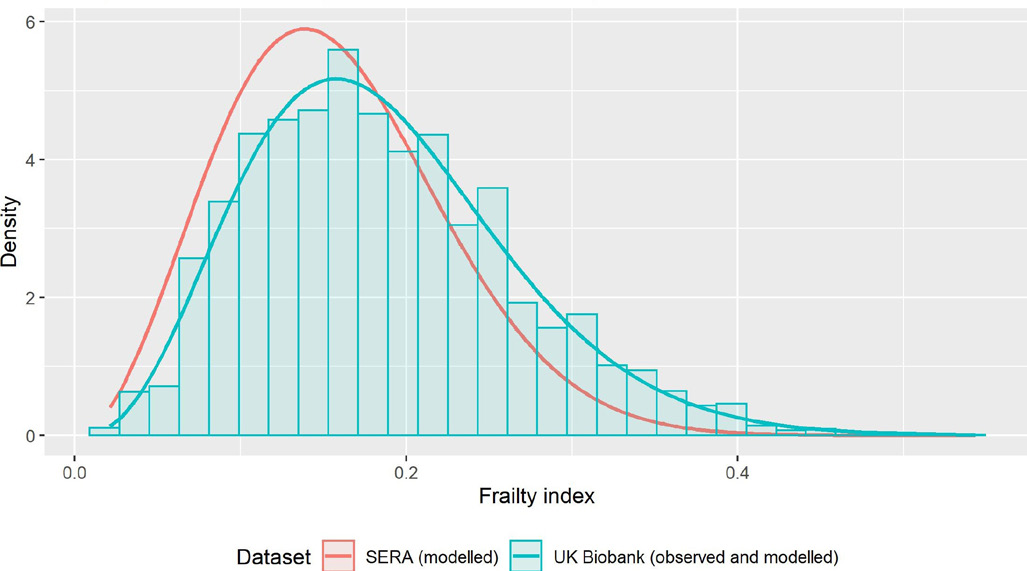
Frailty index distribution (UK Biobank and SERA). This figure shows the distribution of the frailty index in UK Biobank participants (blue bars indicating observed values, blue line showing fitted distribution) and sera participants (red line showing fitted distribution only—observed values analysed within a secure safe-haven and not exported). SERA, Scottish Early Rheumatoid Arthritis.

The mean frailty index was 0.16 in SERA and 0.19 in UK Biobank. The distribution of the frailty index in each of the datasets is shown in figure 1. In SERA, 12.1% of participants had moderate frailty, with 0.2% having severe frailty. The prevalence was higher in UK Biobank, with 714 (20%) participants having moderate and 109 (3%) having severe frailty. All SERA participants had sufficient data to calculate the frailty index. In UK Biobank, 8 participants were excluded due to missing data for >5% of deficits.

Using the frailty phenotype, 781 (23%) of UK Biobank participants met the criteria for frailty, while 1775 (53.1%) were classified as prefrail (compared with 3% and 38%, respectively, in the cohort as a whole).^3^ 44.7% (349/781) participants identified as frail were also moderate or severely frailty by the frailty index criteria. Data for one or more criteria were missing for 262 (7.2%) people with RA (compared with 2% missing data for the cohort as a whole). Hand-grip strength was the most commonly missing variable. Descriptive statistics of participants with missing data are shown in online supplemental appendix.

#### Frailty and disease activity (sera only)

**Figure 2:**
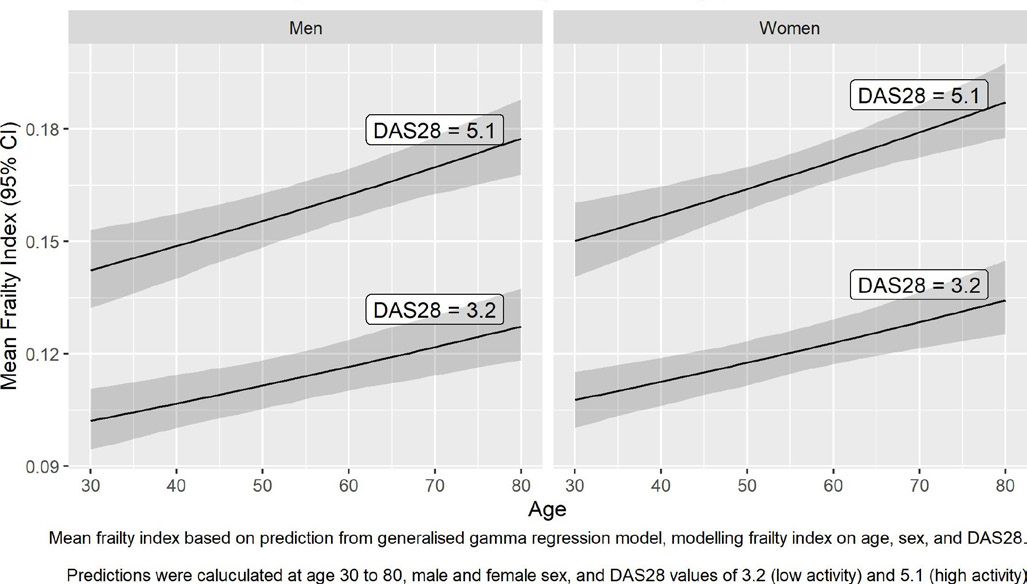
Modelled relationship between frailty index, age, sex and DAS28 in SERA. This figure shows the predicted mean frailty index, based on generalised gamma regression models fitted to the sera dataset, according to age (modelled within range 30–80 years), sex (male and female) and DAS28 (modelled at 3.2 indicating mild disease and 5.1 indicating active disease). Lines indicate point estimates for the mean frailty index, and shaded areas represent 95% CI. DAS28, Disease Activity Score in 28 joints; SERA, Scottish Early Rheumatoid Arthritis.

The modelled relationship between frailty and age, sex and DAS28 in SERA is shown in figure 2. Mean frailty index increased with age, was higher in women than in men, and was higher with more active disease.

#### Frailty and outcomes: serial follow-up in sera

**Figure 3:**
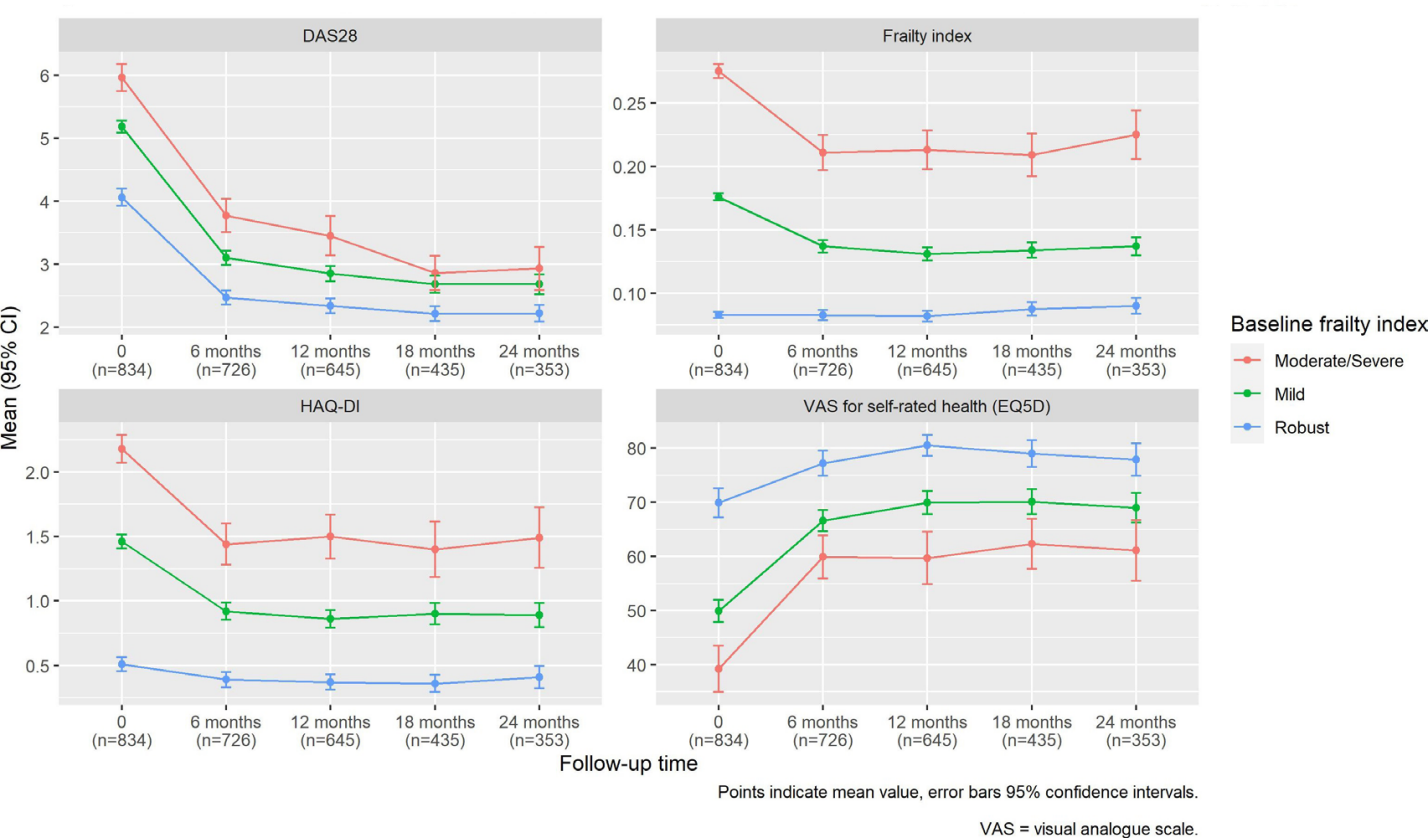
Change in disease activity, frailty index, physical function and self-rated health over 2 years follow-up in SERA. Points indicate mean values for DAS28, frailty index, HAQ-DI and self-rated health, respectively. Error bars indicate 95% CIs. Results are stratified by frailty status at baseline (robust, mild, or moderate/severe) based on the frailty index. DAS28, Disease Activity Score in 28 joints; HAQ-DI, Health Assessment Questionnaire- Disability Index; SERA, Scottish Early Rheumatoid Arthritis.

The change in mean frailty index in SERA over 2-year follow-up is shown in figure 3, along with mean DAS28, HAQ-DI and self-rated health. Data for each measure was available for 834 participants, and this fell to 726, 645, 435 and 353 participants at 6, 12, 18 and 24 months, respectively. However, mean baseline frailty index values were similar between participants with and without missing follow-up data (eg, 0.157 and 0.156 for those with and without missing data at 1 year). Mean frailty index, mean DAS28 and mean HAQ-DI fell after the initial baseline assessment and commencement of DMARD treatment, with improvement in self-rated health. This improvement in mean frailty index reflected an reduction in the overall prevalence of each of the functional measures that were reassessed, but not the laboratory values in the index (which did not substantially change) or comorbidities (which were not reassessed and therefore reflect baseline comorbidity prevalence). However, after 2 years follow-up, HAQ-DI scores, poor self-rated health and, to a lesser extent, disease activity were higher at the group level in participants with mild or moderate/severe frailty at baseline compared with participants who were robust at baseline (figure 3). Of the 109 people who were moderately or severely frail at baseline, 36 (33%) improved to mildly frail and 14 (13%) transitioned to a robust state in the first 6 months of follow-up. Despite these improvements, the mean frailty index at 2 years follow-up among those who were moderately or severely frail at baseline remained significantly higher than participants who were mildly frail or robust at baseline. This indicates that the frailty index is dynamic in early RA and fell concurrently with treatment and improvements in disease activity, physical function and self-rated health. However, despite these improvements, participants with a higher baseline frailty index tended to have a higher frailty index, higher disease activity, poorer physical function and poorer self-rated health through 2 years follow-up compared with participants with a lower baseline frailty index.

#### Frailty and outcomes: linked healthcare data

Associations between frailty and mortality and hospitalisation outcomes are shown in table 2. In both SERA and UK Biobank, moderate/severe frailty (measured using the frailty index) was associated with a higher risk of both all-cause mortality and unscheduled hospitalisation in models adjusted for age, sex and socioeconomic status (model 1), plus smoking and alcohol intake (model 2) and, in SERA only, after additionally adjusting for DAS28. In UK Biobank, mild frailty was also associated with greater risk of mortality and hospitalisation, but in SERA the CI for these estimates included the null. In the sensitivity analysis in SERA, the effect of frailty on both outcomes was similar using the time-varying model compared with using baseline values only.

**Table 2:** Association between frailty and clinical outcomes (all-cause mortality and hospitalisation)

Frailty level	N	All-cause mortality				Unscheduled hospitalisation			
		Events (N)	Model 1 HR (95% CI)	Model 2 HR (95% CI)	Model 3 HR (95% CI)	Events (N)	Model 1 IRR (95% CI)	Model 2 IRR (95% CI)	Model 3 IRR (95% CI)
SERA: Frailty index (three levels)*									
Robust	303	8	ref	ref	ref	152	ref	ref	ref
Mild	487	28	1.83 (0.83 to 4.02)	1.74 (0.79 to 4.29)	1.73 (0.7 to 4.29)	416	1.55 (1.17 to 2.64)	1.47 (1.12 to 1.94)	1.29 (0.93 to 1.77)
Moderate/severe	109	17	3.99 (1.7 to 9.35)	4.41 (1.85 to 10.49)	4.14 (1.49 to 11.51)	189	3.05 (2.09 to 4.47)	2.88 (1.97 to 4.20)	2.27 (1.45 to 3.57)
UK Biobank: Frailty index (three levels)*									
Robust	773	79	ref	ref		618	ref	ref	
Mild	2001	279	1.39 (1.08 to 1.79)	1.36 (1.05 to 1.76)		2520	1.68 (1.44 to 1.97)	1.65 (1.41 to 1.93)	
Moderate/severe	823	158	1.84 (1.4 to 2.43)	1.68 (1.26 to 2.13)		1827	3.13 (2.62 to 3.74)	2.74 (2.29 to 3.29)	
UK Biobank: Frailty index (four levels)									
Robust	773	79	ref	ref		618	ref	ref	
Mild	2001	279	1.39 (1.09 to 1.79)	1.36 (1.05 to 1.76)		2520	1.64 (1.41 to 1.92)	1.65 (1.41 to 1.93)	
Moderate	714	130	1.73 (1.3 to 2.3)	1.59 (1.19 to 2.13)		1443	2.54 (2.11 to 3.05)	2.47 (2.05 to 2.98)	
Severe	109	28	2.75 (1.78 to 4.27)	2.33 (1.49 to 3.64)		384	5 (3.58 to 6.99)	4.8 (3.43 to 6.73)	
UK Biobank: Frailty phenotype									
Robust	788	68	ref	ref		630	ref	ref	
Prefrail	1775	224	1.45 (1.11 to 1.91)	1.37 (1.04 to 1.81)		2209	1.57 (1.34 to 1.84)	1.52 (1.29 to 1.78)	
Frail	781	158	2.54 (1.9 to 3.93)	2.30 (1.71 to 3.10)		1486	2.47 (2.07 to 2.99)	2.38 (1.97 to 2.87)	

Model 1: adjusted for age, sex and socioeconomic status.

Model 2: adjusted for age, sex, socioeconomic status, smoking and alcohol intake.

Model 3: adjusted for age, sex, socioeconomic status, smoking, alcohol intake and DAS28 (SERA only).

*Due to the small number of SERA participants in the severe frailty category (0.2%) these were collapsed into moderate/severe for analysis of SERA. For UK Biobank, the frailty index was analysed using 4-levels (robust, mild, moderate, severe) as prespecified and then using 3-levels (robust, mild, moderate/severe) to mirror the analysis of SERA.

DAS28, Disease Activity Score in 28 joints; IRR, incidence rate ratio; SERA, Scottish Early Rheumatoid Arthritis.

Analyses of the frailty phenotype (UK Biobank only) demonstrated a greater risk of both mortality and hospitalisation associated with both prefrailty and frailty.

## Discussion

Frailty is common in both new onset and established RA. In SERA participants with early RA and in UK Biobank participants with established RA moderate/severe frailty was associated with greater risk of hospitalisation and mortality. In people with early RA, higher baseline frailty index was associated with greater disease activity, functional impairment and poorer self-rated health. The frailty index was dynamic in early RA and as mean disease activity fell with initiation of treatment, so too did the mean frailty index. In SERA, the association between frailty and mortality and hospitalisation remained significant after adjustment for disease activity as well as sociodemographic factors. Frailty is therefore a clinically and prognostically significant marker in RA, although the degree of frailty is likely to fluctuate over time, particularly where it is driven by active RA.

This is the first study to assess frailty in people with early RA (at the point of specialist diagnosis). It is also the first study to assess changes in frailty status over time in RA, demonstrating that frailty in early RA can, at least for some people, improve significantly. This change is likely to reflect an improvement in functional impairment with the initiation of disease modifying treatment. Our hypothesis that improvements in the frailty index are driven by reductions in disease activity and improvements in physical impairment are consistent with previous cross sectional studies showing associations between frailty (although identified using different measures) and both higher disease activity and higher HAQ-DI scores.^4 6 24–30^ It would also explain the higher prevalence of frailty observed in randomised controlled trials for RA,^20^ as high disease activity is typically an explicit requirement for inclusion in these trials.

Our findings indicate that frailty has prognostic significance beyond that of high disease activity. Frailty was associated with all-cause mortality and hospitalisation after adjustment for DAS28. This is consistent with literature on frailty in general populations as well as other long-term conditions.^1 31–33^ Although physical impairment and self-rated health improved after initial diagnosis, participants with moderate frailty at baseline had significantly higher HAQ-DI scores and poorer self-rated health at 2 years follow-up than robust participants or those with mild baseline frailty, despite larger reductions in DAS28 from baseline levels. Our findings also show that while disease activity continues to gradually decline over 2 years on a group level, initial improvements in frailty, HAQ-DI and self-rated health plateaued or worsened over this period. This is consistent with previous observations from SERA, in which psychosocial baseline factors (such as functional disability, depression and unemployment) were more predictive of functional status at 1 year than more traditionally used clinical markers such as disease activity, and supports calls for broad psychosocial factors beyond disease activity to be actively considered when assessing the impact of RA.^15^

Mean frailty index values were higher in UK Biobank than in SERA. This may reflect longer disease duration in UK Biobank participants. Previous studies have shown associations between frailty and duration of RA, however this has not been observed consistently across all studies.^25 28–30^ Another possible explanation is differences in the variables included within the respective frailty indices. While there is no specific set of variables that should be included in a frailty index, and these usually vary between datasets, it is possible that differences in the available variables influenced the distribution of frailty. Both datasets included a similar range of comorbidities, however SERA included more measures of functional impairment (eg, difficulty dressing, climbing stairs) than UK Biobank.

Our findings indicate that frailty may be a useful measure to identify people at greater risk of mortality, hospitalisation, and with greater functional limitation. However, given the close relationship between disease activity and frailty over time, care should be taken in applying a ‘label’ of frailty to people living with RA. The utility of identifying frailty in RA would depend on the intended purpose of the assessment. If frailty is used to identify people who may benefit from a broad, multidisciplinary assessment of health needs, this may be beneficial.^34^ Such an assessment should include identification of reversible factors including, but not limited to, active RA, treatment of which might ameliorate frailty. However, without such an assessment, invoking frailty in the context of inflammatory conditions such as RA may inappropriately identify patients as frail and bias future assessments or interactions with healthcare professionals.

It is important for future research to explore longitudinal trends in frailty, including its correlation with other measures (such as HAQ-DI and quality of life) as well as which factors within the frailty construct are most amenable to change or intervention. The development of frailty is recognised to be multifactorial.^35 36^ There may be multiple subtypes of frailty in RA: those for whom deficits leading to the identification of frailty are driven by active disease, and others for whom it is the result of other comorbidities, age-related decline in physiological function, or other factors. The trajectory, prognostic significance and appropriate response to frailty may differ in each of these situations. It will also be important to explore how frailty in the context of RA differs from other measures, such as multimorbidity, which are also associated with increased mortality risk but have a different conceptual basis.^13 37^

This study is larger than previous studies of frailty in RA, and draws on two independent data sources, each with different strengths. We compared two frailty measures, although each was adapted to available variables. Linkage to national hospital and mortality registers allowed reliable assessment of outcomes. However, both datasets had limitations in the variables available. SERA lacked any assessment of sensory function (eg, vision, hearing) and had relatively few biochemical variables. UK Biobank, in contrast, has few measures of physical function. In SERA, some of these were identified from the HAQ-DI. Although this is consistent with previous applications of the frailty index method, the recognised floor effect of the HAQ-DI may limit the responsiveness of the frailty index to change.^38^ It also means that the reduction in frailty following initiation of treatment is perhaps not surprising, as HAQ-DI is recognised to be responsive to treatment. In assessing the frailty index over SERA follow-up, we did not have any repeated assessment of comorbidities, and therefore had to assume baseline comorbidity status. It is possible that, for some participants, comorbidities may have changed over the 2 years follow-up which would have influenced the frailty index. Participants with RA in SERA were identified using the well-established ACR/EULAR criteria in people attending specialist rheumatology clinics, however in UK Biobank we had to rely on diagnostic codes from routine healthcare data being applied to a population-based cohort. The latter may have resulted in some misclassification. UK Biobank is also recognised to be unrepresentative of the general population, being more affluent and including more people of predominantly White ethnicity than the general UK population. There is also potential for survival bias when assessing UK Biobank participants with RA, as participants were not recruited at the point of diagnosis. People with RA and more severe frailty may be more likely to die prior to recruitment and therefore not be included in UK Biobank. Analyses of UK Biobank are also susceptible to collider bias. For example if people with either more severe RA or severe frailty were less likely to volunteer for UK Biobank (eg, due to greater functional limitation) this could bias estimates of the association between frailty and RA, as well as the relationship between frailty and adverse outcomes in people with RA. A recent analysis of multimorbidity showed that UK Biobank may underestimate associations between higher long-term condition counts and mortality or hospitalisation.^39^ The same may be true of frailty in this context, particularly as long-term conditions contribute heavily to the frailty index. Finally, our analysis of the frailty phenotype was limited to UK Biobank (as grip strength and walking speed were not assessed in SERA) and analysis of disease activity and change in frailty status was limited to SERA. As a result, not all analyses could be replicated in both datasets. Furthermore, there was more missing data for the frailty phenotype (particularly grip strength) in UK Biobank participants with RA compared with the cohort as a whole. It is possible that those with more active disease, pain or functional limitation were more likely to have missing data, which could bias the results.

Frailty is a common and prognostically significant factor in RA, however measured. Active RA is likely to drive at least some of the identification of frailty, however, in early RA frailty may be partially reversible through treatment. Therefore a label of ‘frailty’ should not be applied in early or active RA without reassessment following appropriate treatment and optimisation of RA activity. Frailty identification may be valuable in RA, however should be done with caution and only where identification of reversible factors, broad assessment of health needs and follow-up with reassessment are part of the clinical management.

## Data Availability

Data may be obtained from a third party and are not publicly available. The UK Biobank data that support the findings of this study are available from the UK Biobank (www.ukbiobank.ac.uk), subject to approval by UK Biobank.
