# Supplementary material for: Frailty in rheumatoid arthritis and its relationship with disease activity, hospitalisation and mortality: a longitudinal analysis of the Scottish Early Rheumatoid Arthritis cohort and UK Biobank
Source: RMD Open. 2022 Mar 15;8(1):e002111. doi: 10.1136/rmdopen-2021-002111 (PMC8928366; doi:10.1136/rmdopen-2021-002111)
Supplement: Supplementary data [file rmdopen-2021-002111supp001.pdf]

## SERA frailty index deficits

| Deficit | Source | Coding |
|---|---|---|
| Alcohol problems | Medical history | Present = 1, absent = 0 |
| Anxiety | Medical history | Present = 1, absent = 0 |
| Asthma | Medical history | Present = 1, absent = 0 |
| Atrial fibrillation | Medical history | Present = 1, absent = 0 |
| Bronchiectasis | Medical history | Present = 1, absent = 0 |
| Cancer | Medical history | Present = 1, absent = 0 |
| Coronary heart disease | Medical history | Present = 1, absent = 0 |
| Chronic kidney disease | Medical history | Present = 1, absent = 0 |
| Chronic liver disease | Medical history | Present = 1, absent = 0 |
| COPD | Medical history | Present = 1, absent = 0 |
| Depression | Medical history | Present = 1, absent = 0 |
| Diabetes | Medical history | Present = 1, absent = 0 |
| Diverticular disease | Medical history | Present = 1, absent = 0 |
| Dyspepsia | Medical history | Present = 1, absent = 0 |
| Epilepsy | Medical history | Present = 1, absent = 0 |
| Glaucoma | Medical history | Present = 1, absent = 0 |
| Heart failure | Medical history | Present = 1, absent = 0 |
| Hypertension | Medical history | Present = 1, absent = 0 |
| Osteoporosis | Medical history | Present = 1, absent = 0 |
| Chronic pain | Medical history | Present = 1, absent = 0 |
| Parkinson’s disease | Medical history | Present = 1, absent = 0 |
| Pernicious anaemia | Medical history | Present = 1, absent = 0 |
| Peripheral vascular disease | Medical history | Present = 1, absent = 0 |
| Schizophrenia | Medical history | Present = 1, absent = 0 |
| Stroke or TIA | Medical history | Present = 1, absent = 0 |
| Thyroid disease | Medical history | Present = 1, absent = 0 |
| Difficulty getting out of bed | HAQ-DI | Severe difficulty/unable = 1, some difficulty = 0.5, no difficulty = 0 |
| Difficulty with household chores | HAQ-DI | Severe difficulty/unable = 1, some difficulty = 0.5, no difficulty = 0 |
| Difficulty climbing stairs | HAQ-DI | Severe difficulty/unable = 1, some difficulty = 0.5, no difficulty = 0 |
| Difficulty with shopping (groceries) | HAQ-DI | Severe difficulty/unable = 1, some difficulty = 0.5, no difficulty = 0 |
| Difficult standing | HAQ-DI | Severe difficulty/unable = 1, some difficulty = 0.5, no difficulty = 0 |
| Difficulty with toilet | HAQ-DI | Severe difficulty/unable = 1, some difficulty = 0.5, no difficulty = 0 |

| | | |
|---|---|---|
| Limited mobility | EQ5D-1 | Severe difficulty/unable = 1, some difficulty = 0.5, no difficulty = 0 |
| Difficulty with self-care | EQ5D-2 | Severe difficulty/unable = 1, some difficulty = 0.5, no difficulty = 0 |
| Limited in usual activities | EQ5D-3 | Severe difficulty/unable = 1, some difficulty = 0.5, no difficulty = 0 |
| Pain | EQ5D-4 | Severe difficulty/unable = 1, some difficulty = 0.5, no difficulty = 0 |
| Anxiety | EQ5D-5 | Severe difficulty/unable = 1, some difficulty = 0.5, no difficulty = 0 |
| eGFR | baseline laboratory measures | <30 = 1, <60 = 0.5, >60 = 0 |
| Haemoblobin | baseline laboratory measures | <115 = 1 (men), <110 = 1 (women) |
| Platelets | baseline laboratory measures | <150 = 1, >150 = 0 |

## UK Biobank frailty index deficits

| **Deficit** | **Coding** |
|---|---|
| Glaucoma | Categorised 0/1 |
| Cataracts | Categorised 0/1 |
| Hearing difficulty | Categorised 0/1 |
| Migraine | Categorised 0/1 |
| Dental problems | Categorised 0/1 for none vs. any |
| Self-rated health | 0 – excellent; 0.25 – good; 0.5 - fair, 1 - poor |
| Fatigue: frequency of tiredness / lethargy in last two weeks | 0, 0.25, 0.5, 1, respectively |
| Sleep: experience of sleeplessness/insomnia | Categorised 0, 0.5, 1, respectively |
| Depressed feelings: frequency in last two weeks | 0 – not at all, 0.5 – several days, 0.75 -- more than half, 1 – nearly every day |
| Self-described nervous personality | Categorised 0/1 |
| Severe anxiety/ panic attacks | Categorised 0/1 |
| Common to feel loneliness | Categorised 0/1 |
| Sense of misery (ever/never) | Categorised 0/1 |
| Infirmity: long-standing illness or disability | Categorised 0/1 |
| Falls in last year | 0 - no fall, 0.5 - one fall, 1 - more than one fall |
| Fractures/broken bones in last five years | Categorised 0/1 |
| Diabetes | Categorised 0/1 |
| Myocardial infarction | Categorised 0/1 |
| Angina | Categorised 0/1 |
| Stroke | Categorised 0/1 |

| | |
|---|---|
| High blood pressure | Categorised 0/1 |
| Hypothyroidism | Categorised 0/1 |
| Deep-vein thrombosis | Categorised 0/1 |
| High cholesterol | Categorised 0/1 |
| Breathing: wheeze in last year | Categorised 0/1 |
| Pneumonia | Categorised 0/1 |
| Chronic bronchitis/emphysema | Categorised 0/1 |
| Asthma | Categorised 0/1 |
| Rheumatoid arthritis | Categorised 0/1 |
| Osteoarthritis | Categorised 0/1 |
| Gout | Categorised 0/1 |
| Osteoporosis | Categorised 0/1 |
| Hayfever, allergic rhinitis or eczema | Categorised 0/1 |
| Psoriasis | Categorised 0/1 |
| Any cancer diagnosis | Categorised 0/1 |
| Multiple cancers diagnosed (number reported) | Categorised 0/1 |
| Chest pain | Categorised 0/1 |
| Head and/or neck pain | Categorised 0/1 |
| Back pain | Categorised 0/1 |
| Stomach/abdominal pain | Categorised 0/1 |
| Hip pain | Categorised 0/1 |
| Knee pain | Categorised 0/1 |
| Whole-body pain | Categorised 0/1 |
| Facial pain | Categorised 0/1 |
| Sciatica | Categorised 0/1 |
| Gastric reflux | Categorised 0/1 |
| Hiatus hernia | Categorised 0/1 |
| Gall stones | Categorised 0/1 |
| Diverticulitis | Categorised 0/1 |

# UK Biobank frailty phenotype – comparison of participants with and without missing data

| | Complete data (frailty phenotype) | Missing data (frailty phenotype) |
|---|---|---|
| Total | 3344 | 262 |
| Mean age (sd) | 59.4 (7.1) | 60.4 (6.7) |
| Male (%) | 998 (29.8%) | 65 (24.8%) |
| Female (%) | 2346 (70.2%) | 196 (74.8%) |
| Mean frailty index (sd) | 0.18 (0.08) | 0.20 (0.08) |
